# A Big Role for microRNAs in Gestational Diabetes Mellitus

**DOI:** 10.3389/fendo.2022.892587

**Published:** 2022-07-25

**Authors:** Matladi Masete, Stephanie Dias, Nompumelelo Malaza, Sumaiya Adam, Carmen Pheiffer

**Affiliations:** ^1^ Biomedical Research and Innovation Platform, South African Medical Research Council, Cape Town, South Africa; ^2^ Department of Obstetrics and Gynaecology, Faculty of Health Sciences, University of Pretoria, Pretoria, South Africa; ^3^ Center for Cardio-Metabolic Research in Africa (CARMA), Division of Medical Physiology, Faculty of Health Sciences, Stellenbosch University, Cape Town, South Africa

**Keywords:** microRNAs, pregnancy, type 1 diabetes mellitus, type 2 diabetes mellitus, gestational diabetes mellitus

## Abstract

Maternal diabetes is associated with pregnancy complications and poses a serious health risk to both mother and child. Growing evidence suggests that pregnancy complications are more frequent and severe in pregnant women with pregestational type 1 diabetes mellitus (T1DM) and type 2 diabetes mellitus (T2DM) compared to women with gestational diabetes mellitus (GDM). Elucidating the pathophysiological mechanisms that underlie the different types of maternal diabetes may lead to targeted strategies to prevent or reduce pregnancy complications. In recent years, microRNAs (miRNAs), one of the most common epigenetic mechanisms, have emerged as key players in the pathophysiology of pregnancy-related disorders including diabetes. This review aims to provide an update on the status of miRNA profiling in pregnancies complicated by maternal diabetes. Four databases, Pubmed, Web of Science, EBSCOhost, and Scopus were searched to identify studies that profiled miRNAs during maternal diabetes. A total of 1800 articles were identified, of which 53 are included in this review. All studies profiled miRNAs during GDM, with no studies on miRNA profiling during pregestational T1DM and T2DM identified. Studies on GDM were mainly focused on the potential of miRNAs to serve as predictive or diagnostic biomarkers. This review highlights the lack of miRNA profiling in pregnancies complicated by T1DM and T2DM and identifies the need for miRNA profiling in all types of maternal diabetes. Such studies could contribute to our understanding of the mechanisms that link maternal diabetes type with pregnancy complications.

## Introduction

Maternal diabetes is associated with an increased risk of pregnancy complications and is a significant cause of morbidity for both mother and child (1–3). The prevalence of diabetes during pregnancy is increasing globally, paralleling the obesity and type 2 diabetes mellitus (T2DM) epidemics (4). According to recent estimates, ~16.7% of live births (21.1 million) are associated with maternal diabetes, of which 80.3% are due to gestational diabetes mellitus (GDM), 10.6% due to pre-existing type 1 diabetes mellitus (T1DM) or T2DM, and 9.1% due to T1DM and T2DM first detected in pregnancy (5). All types of maternal diabetes are associated pregnancy complications, with several studies reporting that the frequency and severity of adverse pregnancy outcomes are related with the degree of hyperglycaemia (6, 7). Women with pregestational T1DM and T2DM have a higher risk of pregnancy complications including fetal and neonatal loss, congenital malformations, preterm delivery, macrosomia, preeclampsia and caesarean deliveries, compared to women with GDM (8, 9). The more severe effects of pregestational diabetes compared to GDM are most likely attributed to the pre-conceptual hyperglycaemic environment, longer intrauterine exposure to hyperglycaemia, and the different pathophysiological mechanisms that underlie the different types of maternal diabetes (10, 11).

MiRNAs are short, highly conserved, non-coding RNA molecules that are approximately 22 nucleotides in length. They were first identified in *Caenorhabditis elegans* in 1993 (12) and have emerged as powerful epigenetic mediators of diverse biological processes including development, proliferation, differentiation, apoptosis and metabolism (13). To date over 2 500 miRNAs have been identified in humans (14, 15), which together regulate ~ 60% of genes in the genome (Zhang and Wang, 2017). MiRNAs regulate gene expression through post-transcriptional mechanisms, by binding to the 3’ untranslated region (UTR) of messenger RNA (mRNA) and inducing degradation or by translational repression of the mRNA transcript (16). Furthermore, recent studies have proposed an important role for circulating miRNAs in cell-to-cell communication, suggesting that these extracellular miRNAs may similarly regulate biological processes (17, 18). The dysregulated expression of miRNAs is associated with the development of metabolic disease and conditions including cancer, obesity, T2DM and cardiovascular disease (19; 20).

In recent years, miRNAs have been identified as key regulators of metabolic adaptation during pregnancy (21–23). They regulate several biological processes that are critical during pregnancy and may reflect the physiological state of the pregnancy and fetal development. A growing body of evidence have reported on the association between maternal miRNAs and pregnancy complications, including placental weight (24), placental abruption (25), placental previa (26), preeclampsia and gestational hypertension (27), and intrauterine growth restriction (28), macrosomia (29) and GDM (30). Therefore, miRNA profiling may aid in elucidating the pathophysiological mechanisms that underlie the different types of maternal diabetes. This review aims to provide an update on the status of miRNA profiling in pregnancies complicated by maternal diabetes. Four databases, Pubmed, Web of Science, EBSCOhost, and Scopus, were searched to identify published studies reporting miRNA profiling during maternal diabetes between the date of inception to January 2022. The search terms “type 1 diabetes”, “type 2 diabetes”, “gestational diabetes mellitus”, “pregestational diabetes”, “maternal diabetes”, “microRNA”, and “pregnancy”, including corresponding synonyms and associated terms for each word were used. Studies were considered eligible if they were original articles, investigated miRNA patterns during maternal diabetes, and if the study was published in English. Reference lists of included studies were also searched to identify other potentially eligible studies.

## Characteristics of included studies

**Figure 1:**
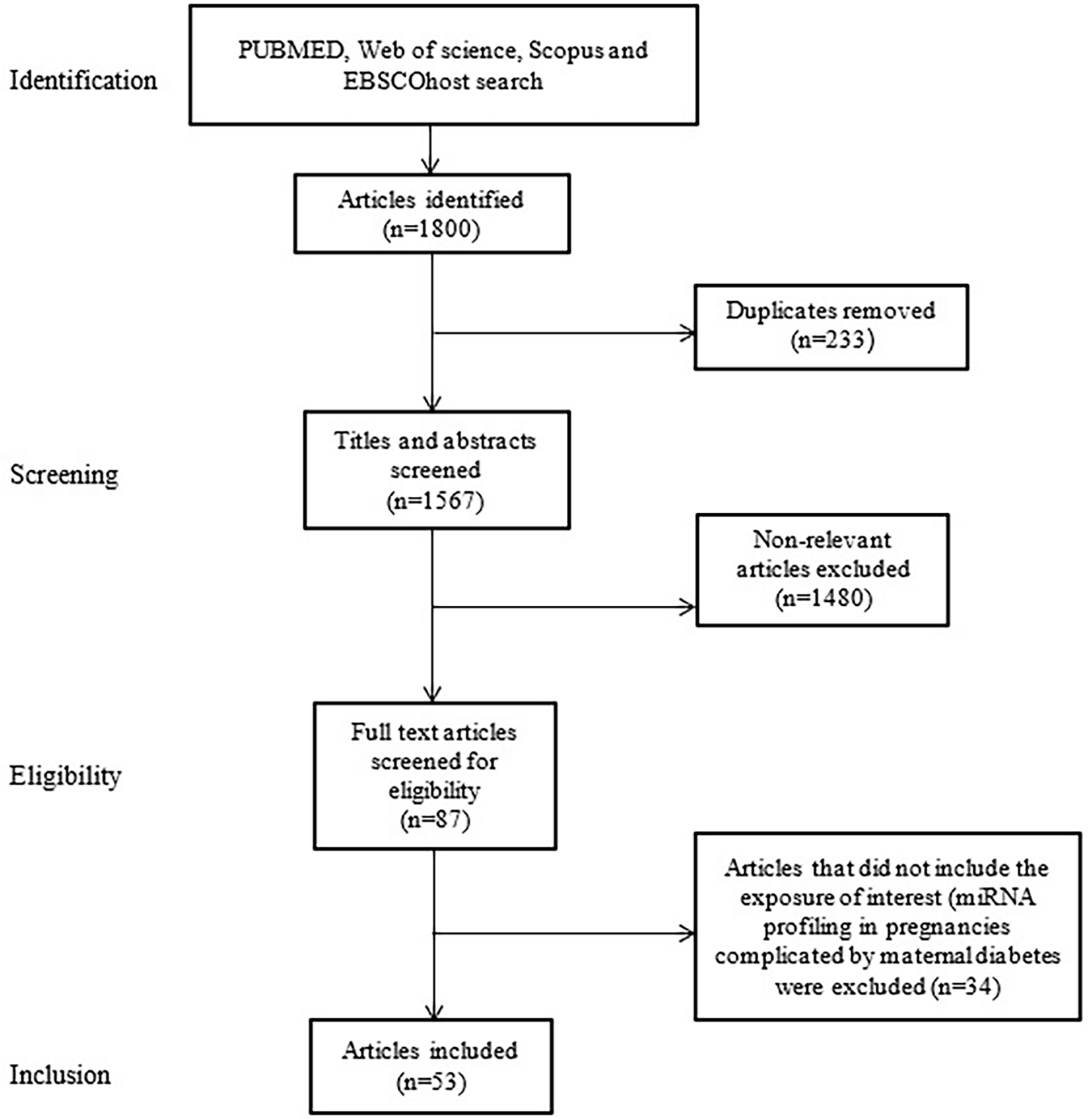
Flow diagram showing selection of studies for inclusion in the review.

A total of 1800 articles were identified from the search strategy, of which 53 met the inclusion criteria and are included in the review (**Figure 1**). The 53 included studies were case-control studies on GDM conducted between 2011 and 2022 (**Table 1**). Studies were conducted across five continents (Africa, Asia, Australia, Europe and North America), with studies conducted in different countries, such as Australian (n = 2), Canada (n = 1), China (n = 33), Estonia (n = 1), Egypt (n = 1), Germany (n = 1), Mexico (n = 3), Iran (n = 1), Italy (n = 2), Italy/Spain (n = 1), South Africa (n = 1), Spain (n = 1), Turkey (n = 3), United States of America (USA) (n = 1) and different places in Europe (n = 1). The sample size of studies ranged from three to 204 women. Studies profiled miRNAs in different biological sources including human umbilical vein endothelial cells (HUVECs) (n = 2), omental adipose tissue (n = 1), plasma (n = 10), placenta/plasma (n = 2), placenta (n = 9), placenta/plasma exosomes/skeletal muscle tissue (n = 1), placenta/whole blood (n = 2), placental-derived mononuclear macrophages (n = 1), serum (n = 16), serum/placenta (n = 1), skeletal muscle tissue (n = 1), urine (n = 1) and whole blood (n = 7). Different measurement platforms and techniques were employed across studies. Studies profiled miRNAs using quantitative real-time PCR (qRT-PCR) with SYBR Green (n = 29), Taqman probes (n = 20), TB Green (n = 1) and qRT-PCR not referenced (NR) (n = 3), for the validation groups. Other studies also used techniques such as miRNA sequencing (n = 5), Taqman Low Density Arrays (n = 1), miRCURY LNA™ Array (n = 2), miScript Array T & B cell activation (n = 1), miScript miRNA Array (n = 2), Dynamic Array Integrated Fluidic Circuits (n = 1), Agilent miRNA Microarrays (n = 1), TaqMan Microarray (n = 2), SurePrint human miRNA Microarray (n = 1), AFFX chip Microarrays (n = 1), NanoString nCounter human miRNA assay (n = 1) and NR (n = 35) for the screening groups. Gestational age at the time of miRNA profiling ranged between 6-40 weeks. Studies used different normalization controls, with the majority using U6 (n = 30) and *C. elegans* miR-39 (n = 12) for circulating miRNAs.

**Table 1:** Studies profiling microRNAs in pregnancies complicated by maternal diabetes.

Author	Country	Study design	Gestational age (weeks)	GDM diagnostic criteria	Sample size	Biologicalsource	Method	Normalization	Outcomes (GDM vs control)
(31)	Egypt	Case-control	Third trimester	NR	GDM = 109 Controls = 103	serum	TaqMan qRT-PCR	cel-miR-39	↑ miR-223 (p<0.001)
(32)	Turkey	Case-control	24-28	ADA, 2010	GDM = 30 Controls = 30	plasma	TaqMan qRT-PCR Dynamic Array Integrated Fluidic Circuits	NR	↑ miR-7-5p (p<0.05), miR-128, miR-129-5p, miR-17-5p, miR-34c-5p, miR-191-5p, miR-29b-3p, miR-384, miR-143-5p, miR-1, miR-342-3p, miR-142-3p; ↓ miR-124-3p, miR-125b-5p, miR-137, miR-139-5p, miR-152, miR-210, miR-24-3p, miR-375, miR-486-5p, miR-9-5p, miR-16-1-3p, miR-26b-3p, miR-214-3p, miR-29a-3p, miR-328, miR-222-5p, miR-126-5p, miR-21-3p, miR-132-3p, miR-198, miR-107, miR-133b, miR-302b-3p, miR-182-5p, miR-197, miR-218.
(33)	China	Case-control	37-40	NR	GDM = 193 Control = 202	placenta	SYBR Green qRT-PCR	U6	↑ miR-98 (p<0.05)
(34)	China	Case-control	24-28	NR	GDM = 85 Controls = 72	plasma	TaqMan qRT-PCR	cel-miR-39, cel-miR-54, cel-miR-238	↑ miR-16-5p (p<0.01), miR-17-5p (p<0.01), miR-20a-5p (p<0.01). **No difference** miR-19a-3p and miR-19b-3p
(35)	China	Case-control	Third trimester	IADPSG, 2010	**Discovery** GDM = 8 Controls = 8 **Validation** GDM = 20 Controls = 18	placenta	**Discovery** miRNA sequencing, **Validation** SYBR Green qRT-PCR	U6	↑ miR-202-5p (p<0.01); ↓ miR-138-5p (p<0.01), miR-210-5p (p<0.05), miR-3158-5p (p<0.01), miR-4732-3p (p<0.05).
(36)	China	Case-control	24-28	Chinese society for Diabetes Mellitus	GDM =12 Controls = 12	Whole blood	TaqMan qRT-PCR	U6	↑ miR-33a-5p (p<0.01)
(37)	Italy	Case-control	24-28	NR	GDM = 22 Controls = 24	HUVECs	SYBR Green qRT-PCR	beta‐actin	↑ miR-101 (p<0.01)
(38)	Canada	Case-control	6-15	Guidelines of the Society of Obstetricians and Gynaecologists of Canada, 2016	GDM = 23 Controls = 46	serum	SYBR Green qRT-PCR	cel-miR-39	↑ miR-520h (p=0.03), miR-1323 (p=0.03), miR-136-5p (p=0.03), miR-342-3p (p=0.008), miR-29a-3p (p=0.03), miR-29b-3p (p=0.04), miR-122-5p (p=0.01), miR-132-3p (p=0.03), miR-182-3p (p=0.01), miR-210-3p (p=0.02). **No difference** miR-494-3p (p=0.10), miR-517-5p (p=0.12), miR-517a-3p, miR-376c-5p, miR-483-3p.
(39)	China	Case-control	NR	NR	GDM = 20 Controls = 20	whole blood	TaqMan qRT-PCR	U6	↓ miR-494 (p<0.01)
(40)	Turkey	Case-control	33 ± 4.1	IADPSG, 2010	GDM = 19 Controls = 28	whole blood	SYBR Green qRT-PCR	U6	↓ miR-21-3p (p = 0.008) **No difference** miR-16-5p, miR155-5p.
(41)	Turkey	Case-control	33 ± 4.1	IADPSG, 2010	GDM = 14 Controls = 27	whole blood	SYBR Green qRT-PCR	U6	↓ miR-155-5p (p=0.04) **No difference** miR-16-5p
(42)	Mexico	Case-control	**First trimester** (8-20) **Second trimester** (24-28) **Third trimester** (32-39)	WHO, 2014	GDM = 27 Controls = 34	urine	TaqMan qRT-PCR	U6	**First trimester** ↑ miR-16-5p (p<0.05), miR-222-3p (p<0.05), miR-516b-5p (p<0.05), miR-517-5p (p<0.05), miR-518-3p (p<0.05) **Second trimester** ↑ miR-16-5p (p=0.009), miR-516b-5p (p=0.043), miR-517-5p (p=0.034), miR-518-3p (p=0.021) **No difference** miR-222-3p (p=0.387), **Third trimester** ↓ miR-16-5p (p<0.01), miR-222-3p (p<0.01), miR-516b-5p (p<0.05), miR-517-5p (p<0.05), miR-518-3p (p<0.01)
(43)	China	Case-control	24-28	IADPSG, 2010	GDM =35 Controls =35	whole blood	TB Green qRT-PCR	U6	↓ miR-4646 (p<0.001), miR-5196 (p<0.001), miR-3679 (p=0.009) **No difference** miR-8061
(44)	China	Case control	37.54 ± 1.31-38.12 ± 1.65	IADPSG, 2010	GDM = 30 Controls = 38	Serum	TaqMan qRT-PCR	U6	↑ miR-377-3p
(45)	Mexico	Case-control	First trimester Second trimester Third trimester	ADA, 2016	**First trimester** GDM = 13 Controls = 12 **Second trimester** GDM = 24 Controls = 24 **Third trimester** GDM = 20 Controls = 16	serum	SYBR Green qRT-PCR	cel-miR-39	**First trimester** ↑ miR-183-5p (p<0.002), miR-200b-3p (p<0.009), miR-125b-5p (p<0.02), miR-1290 (p<0.03) **Second trimester** ↑ miR-183-5p (p=0.03), ↓ miR-200b-3p (p=0.04). **Third trimester** ↑ miR-183 (p<0.0001), ↓ miR-200b-3p (P<0.001).
(46)	China	Case-control	Delivery	Endocrine Society Clinical Practice Guideline, 2013	**Discovery** GDM = 5 Controls = 5 **Validation** GDM = 10 Controls = 10	placenta	**Discovery** Agilent Human miRNA Microarray **Validation** SYBR Green qRT-PCR	U6	↑ miR-508-3p (p<0.01); ↓ miR-27a (p<0.05), miR-9 (p<0.05), miR-137 (p<0.05), miR-92a (p<0.05), miR-33a (p<0.05), miR-30d (p<0.05), miR-362-5p (p<0.05), miR-502-5p (p<0.05). **No difference** miR-148b, miR-10a, miR-370, miR-25, miR-15b.
(47)	China	Case-control	NR	NR	**Screening** GDM = 3 Controls = 3 **Validation** GDM = 15 Controls = 15	placenta/ whole blood	**Screening** miRCURY LNA™ microRNA Array **Validation** qRT-PCR	NR	↓ miR-96 (p<0.01)
(48)	China	Case control	24-28	ADA, 2012	GDM = 110 Controls = 78	Serum	SYBR Green qRT-PCR	U6	↑ miR-1323 (p<0.05)
(49)	Mexico	Case-control	Second-third trimester	IADPSG, 2010	GDM = 18 Controls = 22	serum	TaqMan qRT-PCR	miR-454	↑ miR-9-5p (p=0.03), miR-29a (p=0.01), miR-330 (p=0.004).
(50)	Iran	Case-control	16-19	NR	GDM = 30 Controls = 30	serum	SYBR Green qRT-PCR	U6	↑ miR-135a (p=0.001)
(51)	Australia	Case-control	≥37	ADIPS, 2015 WHO, 2014	GDM = 12 Controls = 12	placenta, plasma exosomes, skeletal muscle tissue	**Screening** Illumina TrueSeq Small RNA kit **Validation** SYBR Green qRT-PCR	U6	**Placenta, plasma exosomes, skeletal muscle tissue:** ↑ hsa-miR-125a-3p (p<0.05), hsa-miR-99b-5p (p<0.05), hsa-miR-197-3p (p<0.05), hsa-miR-22-3p (p<0.05), hsa-miR-224-5p (p<0.05), hsa-miR-27b-3p (p<0.05), hsa-miR-200a-3p (p<0.05), hsa-miR-141-3p (p<0.05). **Placenta:** ↓ hsa-miR-133a-3p (p=0.003) **Plasma exosomes:** ↓hsa-miR-133a-3p (p=0.003) **Skeletal muscle tissue:** ↑hsa-miR-133a-3p (p<0.05)
(52)	Australia	Case control	**Discovery** Early (< 18), mid (22-28) and late (37-40) **Validation** 24-28	**Discovery** ADIPS, 2011 WHO, 2014 **Validation** NDDG, 1979	**Discovery** GDM = 15 Controls = 14 **Validation** GDM = 8 Controls = 14	Plasma	**Discovery** High Output and Illumina NextSeq sequencing **Validation** SYBR Green qRT-PCR	U6	↑ miRNA-92a-3p
(53)	China	Case-control	24-28	ADA, 2010	GDM = 11 Controls = 12	plasma	SYBR Green qRT-PCR	U6	↑ miR-137 (p<0.01)
(54)	Spain	Case-control	26-30	NDDG, 1979	GDM = 31 Controls = 29	serum	TaqMan qRT-PCR	cel-miR-39	↑ miR-330-3p (p = 0.003) **No difference** miR-224-5p, miR-103-3p, miR-206.
(55)	South Africa	Case-control	13-31	IADPSG, 2010	GDM = 28 Controls = 53	serum	SYBR Green qRT-PCR	cel-miR-39	↓ miR-20a-5p (2.7-fold; p=0.038), miR-222-3p (2.6-fold; p=0.027). **No difference** miR-16-5p (1.9-fold; p=0.120), miR-17-5p (2.5-fold; p=0.121), miR-19a-3p (2.6-fold; p=0.056), miR-19b-3p (1.9-fold; p=0.625), miR-29a-3p (2.0-fold; p=0.768), miR-132-3p (2.4-fold; p=0.070).
(56)	Italy	Case-control	24-33	Italian National Health System guidelines, 2011	GDM = 21 Controls = 10	plasma	**Screening** TaqMan microfluidics array **Validation** TaqMan qRT-PCR	miR-374, miR-320	**Screening group** ↑ miR-330-3p (p=0.029), miR-483-5p (2.01-fold; p=0.028). ↓ miR-548c-3p (p=0.028), miR-532-3p (p=0.028). **Validation group** ↑ miR-330-3p (p=0.01) **No difference** miR-548c-3p
(57)	China	Case control	NR	NR	GDM = 25 Controls = 30	Serum	TaqMan qRT-PCR	U6	↑ miR-181d
(58)	China	Case-control	38-39	ADA, 2006	GDM = 13 Controls = 13	omental adipose tissue	AFFX miRNA expression chips microarrays, TaqMan qRT-PCR	miR-16	↑ miR-222 (p<0.01)
(59)	China	Case control	NR	NR	GDM = 20 Controls = 27	HUVECs	SYBR Green qRT-PCR	NR	↑ miR-34b-3p
(60)	Germany	Case-control	24-32	IADPSG, 2010	**Screening** GDM = 8 Controls = 8 **Validation** GDM = 30 Controls 30	whole blood	**Screening** miRNA sequencing **Validation** SYBR Green qRT-PCR	U6	**Screening group** ↑ miR-19a (p=1.48 × 10^-03^), miR-19b (p=5.28 × 10^-03^), miR-142 (p=3.36 × 10^-04^), miR-143 (p=8.72 × 10^-04^), let-7g-5p (p=4.32 × 10^-04^), miR-340 (p=7.06 × 10^-04^). **Validation group** ↑ miR-340 (p=0.03) **No significant change** miR-19a, miR-19b, miR-142, miR-143, let-7g-5p.
(61)	China	Case-control	>37	IADPSG, 2010	GDM = 204 Controls = 202	placenta	TaqMan qRT-PCR	U6	↓ miR-29b (p<0.05)
(62)	Europe	Case-control	<20	IADPSG, 2010 WHO, 2013	GDM = 82 Controls = 41	serum	SYBR Green qRT-PCR	cel-miR-39	↑ miR-29a-3p (p=0.004), miR-134-5p (p=0.046), miR-16-5p (p=0.008).
(63)	Estonia	Case-control	23-31	IADPSG, 2010	GDM = 13 Controls = 9	plasma	**Screening** MiScript miRNA PCR array Human T & B cell activation **Validation** SYBR Green qRT-PCR	cel-miR-39	↑ let-7e-5p (p=0.03), let-7g-5p (p=0.01), miR-100-5p (p=0.04), miR-101-3p (p=0.03), miR-146a-5p (p=0.03), miR-18a-5p (p=0.05), miR-195-5p (p=0.03), miR-222-3p (p=0.03), miR-23b-3p (p=0.02), miR-30b-5p (p=0.04), miR-30c-5p (p=0.02), miR-30d-5p, (p=0.03), miR-342-3p (p=0.04), miR-423-5p (p=0.02), miR-92a-3p (p=0.05).
(64)	USA	Case-control	7-23	ADA, 2004	GDM = 36 Controls = 80	plasma	SYBR Green qRT-PCR	cel-miR-39 and miR-423-3p	↑ miR-155-5p (p=0.028), miR-21-3p (p=0.005), miR-146b-5p (p=0.068). **No difference** miR-517-5p, miR-126-3p, miR-210-3p, miR-222-3p, miR-223-3p, miR-518a-3p, miR-29a-3p.
(65)	China	Case-control	NR	NR	GDM = 48 Controls = 46	placental-derived mononuclear macrophages	SYBR Green qRT-PCR	NR	↑ miR-657 (p<0.001)
(66)	China	Case-control	24-28	NR	GDM = 100 Controls = 100	serum	TaqMan qRT-PCR	U6	↑ miR-19a (4.0-fold; p=0.001), miR-19b (4.77-fold; p=0.02).
(67)	China	Case-control	37-40	NR	GDM = 30 Controls = 29	placenta	TaqMan qRT-PCR	NR	↑ miR-657 (p<0.01)
(68)	China	Case-control	24-28	IADPSG, 2010	GDM = 102 Controls = 102	serum	SYBR Green qRT-PCR	U6	↑ miR-195-5p (p<0.01).
(69)	China	Case control	37-40	NR	GDM = 53 Controls = 46	Plasma	**Screening** SurePrint human miRNA microarray **Validation** SYBR Green qRT-PCR	cel-miR-39	↓ miR-574-5p, miR-3135b
(70)	China	Case control	NR	NR	GDM = 5 Controls = 5	placenta	SYBR Green qRT-PCR	U6	↑ miR-190b
(71)	China	Case control	38.1±1.2- 39.4±1.2	NR	GDM = 26 Controls = 23	Placenta	TaqMan qRT-PCR	NR	↓ miR-6869-5p
(72)	China	Case control	29.10±2.32-32.71±5.26	NR	GDM = 32 Controls = 48	Serum	**Screening** miScript miRNA array **Validation** qRT-PCR	U6	↑ miR-520h
(73)	China	Case-control	16-28	Italian National Health System guidelines, 2011	GDM = 30 Controls = 10	serum	SYBR Green qRT-PCR	U6	↑ miR-330-3p (p<0.001)
(74)	China	Case-control	NR	NR	**Placenta** GDM = 3 Controls = 3 **Whole blood** GDM = 25 Controls = 25	placenta/ whole blood	**Screening** miRCURY LNA™Array **Validation** qRT-PCR	NR	↑ miR-503 (p<0.01).
(75)	Italy/Spain	Case-control	9-12	IADPSG, 2010	GDM = 23 controls = 20	plasma	**Screening** NanoString nCounter human miRNA assay **Validation** TaqMan qRT-PCR	cel-miR-39	↑ miR-23a (p=1.92 × 10^−2^), miR-223 (p=1.42 × 10^−7^). **Validation** ↑ miR-223 (p= 0.009), miR-23a (p=0.03).
(76)	China	Case control	24-28	ADA, 2014	GDM = 123 Controls = 123	Plasma/Placenta	TaqMan qRT-PCR	U6	↓ miR-96-5p
(77)	China	Case control	Placenta 37-41 Blood plasma 26-40	IADPSG, 2010	**Screening** GDM = 3 Controls = 3 **Validation** GDM = 36 Controls = 37	Placenta/plasma	**Screening** NextSeq sequencing **Validation** SYBR Green qRT-PCR	U6	↑ miRNA-144 (p < 0.001) ↓ miRNA-125b (p < 0.001)
(78)	China	Case control	<37	NR	GDM = 166 Controls = 196	Placenta	qRT-PCR	U6	↓ miR-30d-5p
(79)	China	Case-control	16-19	ADA, 2004	**Discovery sample** GDM = 24 Controls = 24 **Internal validation** GDM = 36 Controls = 36 **External validation 1** GDM = 16 Controls = 16 **External validation 2** GDM = 16 Controls = 16	serum	**Discovery** TaqMan Low Density Arrays **Validation** Individual SYBR Green qRT-PCR	**Discovery** U6 cel-miR-39 **Validation** cel-miR-39	**Discovery sample** ↓ miR-132 (p=0.042), miR-29a (p=0.032), miR-222 (p=0.041). **Internal validation** ↓ miR-132 (p=0.034), miR-29a (p=0.045), miR-222 (p=0.016). **External validation 1** ↓ miR-29a (p=0.001), miR-222 (p=0.017). **No difference** miR-132 (p=0.235) **External validation 2** ↓ miR-132 (p=0.001), miR-29a (p=0.001), miR-222 (p=0.001).
(80)	China	Case-control	37-40	NR	GDM = 40 Controls = 40	placenta	TaqMan qRT-PCR	U6	↑ miR-518d (p<0.01)
(81)	China	Case-control	NR	NR	GDM = 30 Controls = 30	whole blood	SYBR Green qRT-PCR	U6	↑ miR-770-5p (p<0.01)
(82)	China	Case-control	24-28	ADA, 2012	GDM = 108 Controls = 50	serum/ placenta	SYBR Green qRT-PCR	U6	↓ miR-132 (p<0.001)
(83)	China	Case-control	16-19	ADA, 2011	GDM = 10 Controls = 10	plasma	**Screening** MiRNA sequencing **Validation** SYBR Green qRT-PCR	miR-221	↑ miR-16-5p (p=5.36 × 10^-11^), miR-17-5p (p=1.10 × 10^-10^), miR-19a-3p (p=6.57 × 10^-43^), miR-19b-3p (p=1.73 × 10^-74^), miR-20a-5p (p=5.27 × 10^-37^).

↑– up-regulation; ↓ – down-regulation; p value and fold regulation reported if given in article; ADA – American Diabetes Association; ADIPS – Australasian Diabetes in Pregnancy Society; GDM – gestational diabetes mellitus; IADPSG – International Association of Diabetes in Pregnancy Study Group; NDD – National Diabetes Data Group; NR – not reported; qRT-PCR – quantitative reverse transcription PCR; WHO – World Health Organisation; Chinese society for Diabetes Mellitus (year not specified); TB Green -Premix Ex TaqII (Tli RNase H Plus); HUVECs – Human umbilical vein endothelial cells.

## Qualitative synthesis of studies

All the included studies profiled miRNAs during GDM with no studies on pregestational T1DM and T2DM identified. A total of 32 miRNAs were assessed in two or more studies and are discussed below. These included miR-9 (n = 3), miR-16 (n = 7), miR-17 (n = 4), miR-19a (n = 5), miR-19b (n = 5), miR-20a (n = 3), miR-21 (n = 3), miR-29a (n = 7), miR-29b (n = 3), miR-30d (n = 3), miR-92a (n = 3), miR-96 (n = 2), miR-125b (n = 2), miR-132 (n = 5), miR-137 (n = 3), miR-142 (n = 2), miR-143 (n = 2), miR-155 (n = 3), miR-195 (n = 2), miR-197 (n = 2), miR-210 (n = 4), miR-222 (n = 7), miR-223 (n = 3), miR-330 (n = 4), miR-342 (n = 3), miR-483 (n = 2), miR-494 (n = 2), miR-517 (n = 3), miR-520h (n = 2), miR-657 (n = 2), miR-1323 (n = 2) and let-7g (n = 2).

Three studies that reported on the expression of miR-9. Of the three studies, one study reported higher levels of miR-9 in the serum of Mexican women with GDM compared to controls (49). In contrast, two studies profiling miRNAs in placental tissue of Chinese women (46) and in plasma samples of Turkish women (32) reported lower expression of miR-9 in women with GDM compared to controls. Of the seven studies reporting on miR-16, three studies demonstrated higher expression in serum and plasma samples of Chinese and European women with GDM compared to controls (34, 62, 83). In contrast, Herrera et al. (42) reported lower expression of miR-16 in urine samples of Mexican women with GDM compared to controls in the third trimester, and higher expression in the first and second trimesters (42). Three studies conducted in Turkey and South Africa reported no difference in miR-16 expression in women with GDM compared to controls (40, 41, 55). Four studies investigated miR-17 during GDM. Of these, three studies reported that miR-17 expression was higher in plasma samples of Chinese and Turkish women with GDM compared to controls (32, 34, 83). In contrast, Pheiffer et al. (55) showed no significant difference in miR-17 expression in the serum of South African women with GDM compared to controls (55). Five studies profiled miR-19a and miR-19b during GDM. Two studies reported that miR-19a and miR-19b expression was higher in serum and plasma samples of Chinese women with GDM compared to pregnant women without GDM (66, 83). However, two studies reported that miR-19a and miR-19b expression did not differ in plasma and serum samples of women with GDM compared to controls (34, 55). Stirm et al. (60) demonstrated higher expression of miR-19a and miR-19b in the whole blood of German women with GDM compared to controls in the screening group, however, this difference was not validated in a larger sample (60).

Three studies profiled miR-20a, of which two studies reported higher expression of miR-20a in Chinese women with GDM when compared to controls (34, 83), while Pheiffer et al. (55) reported lower expression of miR-20a in South African women during GDM compared to controls (55). For miR-21, Wander et al. (64) reported higher expression in plasma samples of American women with GDM compared to controls (64), while two studies reported lower expression of miR-21 in whole blood and plasma samples of Turkish women with GDM compared to controls (32, 40). MiR-29a was investigated in seven studies. Of these, three studies showed higher serum expression of miR-29a during GDM in women from Canada, Mexico, and different regions in Europe (38, 49, 62). Two studies reported lower levels of miR-29a in serum and plasma of Chinese and Turkish women with GDM compared to controls (32, 79), and two studies reported no difference in miR-29a expression in serum and plasma samples of American and South African women with GDM compared to controls (55, 64). Of the three studies that reported on miR-29b expression during GDM, two studies reported higher expression in serum and plasma samples of Canadian and Turkish women with GDM compared to controls (32, 38), while Sun etal. (61) reported lower expression of miR-29b in Chinese women with GDM compared to controls (61).

Three studies investigated miR-30d during GDM. Of these, two studies reported higher expression of miR-30d in plasma and placenta of Estonian and Chinese women with GDM compared to controls (63, 78), while one study reported lower expression in placenta samples of Chinese women with GDM compared to controls (46). Three studies reported on miR-92a during GDM. Of these, two studies reported higher expression in plasma of miR-92a during GDM (52, 63), while Lie at al. (46) reported lower expression in the placenta of Chinese women with GDM compared to controls (46). The two studies that investigated miR-96, both reported lower expression in plasma/placenta/whole blood of Chinese women with GDM compared to controls (47, 76). Two studies reported contradicting results for miR-125b. Lamadrid-Romero et al. (45) reported higher expression of miR-125b in serum samples of Mexican women with GDM compared to controls (45), while Balci et al. (32) reported lower expression in plasma samples of Turkish women with GDM compared to controls. Of the five studies, only one study reported a higher expression of miR-132 in the serum samples of Canadian women (38). Contradictingly, three studies reported a lower expression of miR-132 in serum and plasma samples of Chinese and Turkish women with GDM (32, 79, 82). However, Pheiffer et al. (55) observed no significant change in expression of miR-132 in serum samples of South African women with GDM when compared to controls (55).. All three studies that investigated miR-137 reported lower expression in plasma and placenta samples of Chinese and Turkish women with GDM compared to controls. (32, 46, 53). Two studies reported on the expression of miR-142 and miR-143 during GDM. One study reported higher expression of miR-142 and miR-143 in plasma of Turkish women with GDM compared to controls (32). Stirm et al. (60) reported higher expression of miR-142 and miR-143 in the whole blood of German women with GDM in the screening group, however, these findings were not validated in a larger sample. Of the three studies that investigated miR-155, one study reported higher expression in plasma samples of American women with GDM compared to controls (64). Hocaoglu et al. (40) reported no change in the expression of miR-155 in whole blood of Turkish women with GDM compared to controls (40). However, a more recent study by the same authors reported lower expression of miR-155 in whole blood of Turkish women with GDM compared to controls (41). Both studies that investigated miR-195 reported higher expression in plasma samples of Estonian and Chinese women with GDM compared to controls (63, 68). Contradicting results were reported for the expression miR-197. Nair et al. (51) reported higher expression of miR-197 in placenta, exosomes and skeletal muscle tissue samples of Australian women with GDM compared to controls (51), while Balci et al. (32) reported lower expression of miR-197 in plasma samples of Turkish women with GDM compared to controls (32).

Four studies reported on the expression of miR-210. Of these, one study reported higher levels of miR-210 in serum samples of Canadian women with GDM compared to controls (38), while lower levels of miR-210 was observed in placental and plasma samples of Chinese and Turkish women with GDM (32, 35). Wander at al. observed no difference in miR-210 expression in plasma samples of American women with GDM compared to controls (64). Of the seven studies that reported on miR-222 expression during GDM, two reported higher expression of miR-222 in omental adipose tissue and plasma of Chinese women with GDM compared to controls (58, 63), while three studies observed lower expression of miR-222 in serum of Chinese, South African and Turkish women with GDM compared to controls (32, 55, 79). Wander et al. (64) observed no difference in the expression of miR-222 in plasma of American women with GDM compared to controls (64). Herrera-Van Oostdam et al. (42) demonstrated higher expression of miR-222 in urine samples of Mexican women with GDM compared to controls in the first trimester and observed no significant difference in the second trimester and lower expression in the third trimester (42). Two studies reported increased levels of miR-223 in serum and plasma of women during GDM from Italy/Spain and Egypt (31, 75), however, Wander et al. (64) reported no difference in the expression of miR-223 in plasma of American women with GDM compared to controls (64).

All four of the studies that profiled miR-330 reported higher levels in serum and plasma of Italian, Mexican, Spanish and Turkish women with GDM compared to controls (49, 54, 56, 74). All three studies that profiled miR-342 demonstrated higher expression in serum and plasma of Estonian, Canadian and Turkish women with GDM compared to controls (32, 38, 63). Sebastiani et al. (56) reported higher expression of miR-483 in plasma of Italian women with GDM compared to controls (56), while Gillet et al. (38) showed no difference in expression of miR-483 in serum samples of Canadian women with GDM compared to controls (38). He et al. (39) reported lower expression of miR-494 in whole blood samples of Chinese women with GDM compared to controls (38), while Gillet et al. (39) demonstrated no difference in the expression of miR-494 in serum samples of Canadian women with GDM compared to controls (38). Of the three studies that investigated miR-517, Herrera-Van Oostdam et al. (42) demonstrated higher expression in women with GDM compared to controls in the first and second trimesters but lower expression in the third trimester (42). The other study that profiled miR-517 showed no difference in expression in serum of women with GDM compared to controls (35, 64). Both studies that profiled miR-520h reported higher expression in serum of Canadian and Chinese women with GDM compared to controls (38, 72). Two studies investigated miR-657 during GDM, and both studies reported higher expression in placental and placental-derived mononuclear macrophages of Chinese women with GDM compared to controls (65, 67). Both studies reporting on miR-1323 observed higher levels in the serum of Canadian and Chinese women with GDM compared to controls (38, 48). Two studies reported on the expression of let-7g. Tagoma et al. (63) reported higher expression of let-7g in plasma samples of Estonian women with GDM compared to controls (57). Stirm et al. (60) reported conflicting results on the expression of let-7g. These authors reported higher expression of let-7g in the screening group, however, no difference was observed in the validation group in whole blood samples of German women with GDM compared to controls in the screening group (60)

Other articles included in this review reported differential miRNA expression, yet these miRNAs were identified in single studies only (36, 37, 43, 44, 48, 50, 57, 59, 69, 71, 74, 76, 77, 80, 81).

## Discussion

MiRNA profiling in pregnancies complicated by diabetes may aid in elucidating the pathophysiological mechanisms that underlie T1DM, T2DM, and GDM (21–23, 84). This review provides an update on the status of miRNA profiling in pregnancies complicated by maternal diabetes. The main finding of this review is the lack of studies that have profiled miRNAs in pregnant women with pregestational T1DM and T2DM. All the included studies investigated GDM only. Of these, six miRNAs [miR-195 (n = 2), miR-330 (n = 4), miR-342 (n = 3), miR-520h (n = 2), miR-657 (n = 2) and miR-1323 (n = 2)] were similarly differentially expressed in pregnant women with GDM compared to controls in two or more studies (**Table 2**). The consistency of expression of these miRNAs across diverse populations and gestational ages and using different methodologies and measuring platforms support their candidacy as biomarkers of GDM.

**Table 2:** MicroRNAs upregulated during GDM in two or more studies.

MiRNA	Authors	Country	GA (weeks)	Biologicalsource	Method	Control	Biological mechanisms	Pregnancy Outcomes
miR-195	63; 68; *^85^	China Estonia	23 - 31	serum plasma	miScript miRNA PCR Human T and B cell activation SYBR green qRT-PCR	Cel-miR-39 U6	Fatty acid biosynthesis and metabolism Insulin signaling Glycogen synthesis	Obesity *T2DM
miR-330	56; 49; 54; 73	Italy Mexico Spain China	16 - 30	serum plasma	TaqMan microarray SYBR green qRT-PCR	miR-374 miR-320 miR-454 cel-miR-39 U6	β-cell function Glucose homeostasis Insulin secretion	Caesarean delivery
miR-342	63; 32, 38; *^86^	Estonia Canada Turkey	6 - 31	serum plasma	TaqMan qRT-PCR Dynamic Arrays MiScript miRNA PCR array Human T & B cell activation SYBR green qRT-PCR	cel-miR-39	Fatty acid biosynthesis and metabolism Insulin secretion β-cell development	Obesity *Cardiovascular disease
miR-520h	38, 72; *^28^	Canada China	6 - 40	serum	miScript miRNA array SYBR green qRT-PCR	cel-miR-39 U6	Insulin secretion Inhibit cell viability Promote apoptosis	*Preeclampsia
miR-657	65, 67; *^87^	China	37 - 40	placental-derived mononuclear macrophages placenta	SYBR green qRT-PCR TaqMan qRT-PCR	NR	Macrophage proliferation, migration, and polarization	*T2DM
miR-1323	48, 65; *^88^	Canada China	6 - 40	serum	SYBR green qRT-PCR	cel-miR-39 U6	Insulin secretion Trophoblast function	*Preeclampsia

GDM – gestational diabetes mellitus; T2DM – type 2 diabetes mellitus; NR – not reported; qRT-PCR – quantitative reverse transcription PCR; β-cell- beta cell; GA – gestational weeks

*Pregnancy outcomes were not reference in these articles, however they were discussed in other articles.

Despite our search identifying 53 articles on miRNA profiling during maternal diabetes, none investigated pregestational T1DM and T2DM. Previously, Collares et al. (89) profiled miRNAs in non-pregnant individuals with T1DM and T2DM, and in women with GDM (89). These authors identified several miRNAs that were unique to each diabetes type. Eleven miRNAs, let-7f, let-7g, miR-103, miR-1260, miR-1274a, miR-1274b, miR-130a, miR-150, miR-20b, miR-21 and miR-720 were unique to T1DM. Five miRNAs, miR-140-3p, miR-199a-3p, miR-222, miR-30e and miR-451 were unique to T2DM. Ten miRNAs, miR-101, miR-1180, miR-1268, miR-181a, miR-181d, miR-26a, miR-29a, miR-29c, miR-30b and miR-595 were unique to GDM (89). Specific miRNAs may represent biological markers for each type of diabetes, warranting further investigation as potential mechanisms that underlie the different diabetes types. Collares et al. (89) assessed miRNA expression in both females and males, and included non-pregnant individuals, thus their results do not reflect placental-derived miRNAs and pregnancy pathophysiology. Ibarra et al. (90) profiled miRNAs during pregestational T1DM and T2DM (90). This research was reported as a conference abstract only and was not included in this review. Data from the abstract report that miR-19a, miR-125b, miR-20a and a miRNA on Chr11-134 were unique to placenta samples of women with T1DM and were not expressed in pregnant women with T2DM (90). Our review highlights the lack of studies profiling miRNAs in pregnancies complicated by pregestational T1DM and T2DM. We propose that future studies on miRNA profiling include all types of maternal diabetes, which may contribute to elucidating the different pathophysiological mechanisms that underlie pregestational T1DM and T2DM, and GDM.

Studies on miRNA profiling during GDM were mainly related to biomarker discovery. These studies identified six miRNAs that were consistently expressed at higher levels in serum, plasma, placenta and placental-derived mononuclear macrophages in women with GDM compared to controls in different populations, using different methodologies and measurement platforms, and during different gestational ages. These include miR-195 (n = 2), miR-330 (n = 4), miR-342 (n = 3), miR-520h (n = 2), miR-657 (n = 2) and miR-1323 (n = 2) (**Table 2**). MiR-195 levels were reported to be consistently higher in the serum and plasma samples of women with GDM compared to controls across two studies conducted in China and Estonia using miScript miRNA PCR Human T and B cell activation, and SYBR green qRT-PCR (63, 68). Previous studies observed high levels of miR‐195 associated with fatty acid biosynthesis and metabolism, insulin signaling cascade and glycogen synthesis (63, 85), suggestive of miR-195 candidacy as a biomarker for GDM. Furthermore, upregulation of miR-195 in women with GDM was shown to be associated with the development of T2DM (85) and obesity (63, 68). Interestingly, circulating levels of miR-330 was consistently higher in the serum and plasma samples of women with GDM compared to controls across four studies conducted in Italy, Mexico, Spain and China using TaqMan microarray, TaqMan and SYBR green qRT-PCR (49, 54, 56, 73). MiR-330 regulates genes involved in beta-cell (β-cell) function and glucose homeostasis, suggesting that increased miR-330 expression may lead to impaired β-cell proliferation and insulin secretion (56). Furthermore, upregulation of miR-330 was shown to be associated with caesarean delivery in women with GDM (54, 56). MiR-342 levels were reported to be higher in serum and plasma of Estonian, Canadian and Turkish women with GDM compared to controls using TaqMan qRT-PCR, MiScript miRNA PCR array Human T & B cell activation and SYBR green qRT-PCR (32, 38, 63). MiR-342 has been associated with the regulation of fatty acid biosynthesis and metabolism (63), impaired insulin secretion (38) and β-cell development (32). Furthermore, upregulation of miR-342 in women with GDM was shown to be associated with obesity (63) and cardiovascular disease in children born to mothers with GDM (86). MiR-520h levels were reported to be higher in serum of Canadian and Chinese women with GDM compared to controls using miScript miRNA array and SYBR green qRT-PCR (38, 72). MiR-520h is implicated in impaired insulin secretion in pancreatic β-cells (38), and has been demonstrated to inhibit cell viability and promote apoptosis (72). Furthermore, the upregulation of plasma miR-520h during the first trimester was associated with the onset of preeclampsia (28). MiR-657 levels were reported to be higher in placental-derived mononuclear macrophages and placenta samples of women with GDM in a Chinese population using SYBR green qRT-PCR and TaqMan qRT-PCR (65, 67). MiR-657 regulates inflammation *via* targeting Interleukin-37/Nuclear factor-κB signalling axis, that is responsible for the regulation of inflammatory responses (65). Furthermore, the upregulation of miR-657 was associated with the pathogenesis of T2DM (87). MiR-1323 was expressed at higher levels in serum samples of women with GDM compared to controls in studies conducted in Canada and China using SYBR green qRT-PCR (38, 48). MiR-1323 regulates insulin secretion (38) and trophoblast cell activity crucial for placental cell development (48). MiR-1323 was implicated in patients with preeclampsia (88). MiRNAs that are commonly expressed across diverse populations and gestational ages, biological samples and using different measurement platforms present opportunities as biomarkers for GDM. Although it could be argued that miRNAs offer little advantage over measurement of glucose concentrations, the oral glucose tolerance test, the gold standard for GDM diagnosis, is associated with several disadvantages which include the requirement for fasting, multiple blood draws, and association with nausea, vomiting and bloating, lead to decreased patient compliance (30). Furthermore, as discussed above, these miRNAs have been reported to be associated with adverse pregnancy outcomes, supporting their use as biomarkers to predict pregnancy outcomes.

Findings from this review show heterogenous miRNA expression across studies with a general lack of reproducibility. MiRNA heterogeneity may be attributed to factors such as diet, physical activity, medication use, population differences such as ethnicity, socioeconomic status, environmental factors and viral infections (91–95), and differing gestational ages between women (42). Furthermore, different GDM diagnostic criteria and glucose cut-off values across studies may have also contributed to miRNA variability. Pre-analytical and analytical factors such as sample collection and storage, miRNA isolation procedures, measurement platform, and normalisation methods (96–98) affect miRNA expression analysis. The development of optimized protocols for standardizing sample collection, transport, and storage, as well as miRNA isolation procedures and data analysis for the diversity of technological methods used are important to improve reproducibility across studies. Importantly, the non-specificity of miRNAs is another factor that may limit its clinical applicability. MiRNAs are able to regulate multiple genes across different biological pathways in different diseases (99, 100), therefore, miRNA signatures based on a pool of miRNAs may have more clinical applicability than individual miRNAs. Although rapid technological advances could facilitate the use of miRNAs as inexpensive, point-of-care biomarkers in the future, at present, miRNA profiling during GDM remains inconclusive, largely due to poor reproducibility between studies. Many pre-analytical, analytical and biological challenges must be addressed before miRNAs can become clinically applicable. Although it could be argued that miRNAs offer little advantage over measurement of glucose concentrations, the oral glucose tolerance test, the gold standard for GDM diagnosis, is associated with several disadvantages which include the requirement for fasting, multiple blood draws, and association with nausea, vomiting and bloating, which leads to decreased patient compliance (30). Furthermore, as discussed above, these miRNAs have been reported to be associated with adverse pregnancy outcomes, supporting their use as biomarkers to predict pregnancy outcomes.

## Conclusion and future perspectives

This review highlights the lack of studies profiling miRNA expression in pregnancies complicated by pregestational T1DM and T2DM. Future studies should prioritise miRNA profiling in all types of maternal diabetes, which may aid in identifying the mechanisms that underlie the different types of diabetes during pregnancy. Such studies could contribute to unravelling the link between diabetes type and pregnancy outcomes. Furthermore, this review confirms the growing evidence supporting the potential of miRNAs to serve as biomarkers of GDM. Six miRNAs with similar expression in women with GDM compared to controls in two or more studies, across different populations and gestational ages, using different methodologies and measuring platforms are highlighted. These six miRNAs represent candidates as future GDM biomarkers and should be prioritized in future studies.

## Author Contributions

MM and CP, conceptualization and original draft. MM and NM, literature search and study selection. MM and CP, data extraction. MM, SD, NM, SA, and CP, manuscript writing and approval of the final draft. All authors contributed to the article and approved the submitted version.

## Funding

Research Foundation (NRF) Competitive Programme for Rated Researchers Grant No: 120832 to C Pheiffer. Baseline funding from the South African Medical Research Council (SAMRC) is also acknowledged. Mr Masete is a recipient of the SAMRC Division of Research and Capacity Development internship scholarship funding programme. The content hereof is the sole responsibility of the authors and do not represent the official views of the NRF or SAMRC.

## Conflict of Interest

The authors declare that the research was conducted in the absence of any commercial or financial relationships that could be construed as a potential conflict of interest.

## Publisher’s Note

All claims expressed in this article are solely those of the authors and do not necessarily represent those of their affiliated organizations, or those of the publisher, the editors and the reviewers. Any product that may be evaluated in this article, or claim that may be made by its manufacturer, is not guaranteed or endorsed by the publisher.
